# Examiner trust in applicants to the European Patent Office: country specificities

**DOI:** 10.1007/s11192-018-2894-4

**Published:** 2018-11-19

**Authors:** Joaquín M. Azagra-Caro, Elena M. Tur

**Affiliations:** 1 INGENIO (CSIC-UPV), Universitat Politècnica de València, Camino de Vera s/n, 46022 Valencia, Spain; 2 School of Innovation Sciences, Eindhoven University of Technology, Eindhoven, The Netherlands; 3 Institute of Innovation and Entrepreneurship, School of Business, Law and Economics, University of Gothenburg, Gothenburg, Sweden

**Keywords:** Patent citations, European Patent Office, Patent examiners, Knowledge flows

## Abstract

Indicators based on the probability of applicant citations in patents have been used to emphasize the importance of distinguishing applicant and examiner citations. However, the interpretation of these indicators and of the presence of applicant citations in European Patent Office (EPO) examiner reports is still uncertain. Based on interviews with patent examiners and patent applicants, we develop the idea that applicant citations in EPO examiner reports indicate examiner trust in applicants, and that this trust varies according to national patterns. Using EPO data for over 3,500,000 citations during 1997–2007, we verify that examiner trust in applicants is higher in granted patents. Examiners trust applicants from scientifically or economically strong countries, from member states of the European Patent Organization, and from the same country of the examiners.

## Introduction

Much research makes extensive use of backward citations in patents to build indicators of knowledge flows (Jaffe et al. 1993; Maurseth and Verspagen 2002; Ribeiro et al. 2014; van Raan 2017). Some works emphasized the importance of distinguishing whether the citation was introduced by the applicant or the examiner to avoid biases in the study of knowledge flows (Thompson 2006; Alcácer and Gittelman 2006; Criscuolo and Verspagen 2008; Azagra-Caro et al. 2009).^1^ Later works have argued that each type of citation is useful to analyze different phenomena (Acosta et al. 2013; Barirani et al. 2013; Li et al. 2014; Yasukawa and Kano 2014). However, none discusses the interpretation of the probability of applicant citations.

Alcácer et al.’s (2009) study of the United States Patent and Trademark Office (USPTO) contributes by interpreting applicant citation shares in a patent as indicators of ‘applicant search effort’^2^: a large share of applicant citations in a patent would be an indicator of the applicant’s thorough search of the prior art. The most relevant references would be included, leaving few gaps for the patent examiner to fill. This interpretation is especially relevant to the USPTO because the US patent system imposes a ‘duty of candor’ on applicants which requires them to disclose the entire relevant prior art when filing a patent. At the USPTO, if a third party can prove that the applicant deliberately hid or withheld relevant information, the patent becomes unenforceable. This creates an incentive for applicants to the USPTO to include more citations in their applications, i.e. to put ‘effort’ into prior art search. The European patent system does not impose a duty of candor, so applicants need not include all citations to the literature, and the number of applicant citations is much lower in EPO than in USPTO patents (Criscuolo and Verspagen 2008; Azagra-Caro et al. 2011). EPO applicants include more citations because they judge it to be in their best interests, and not necessarily because they have invested more effort than applicants who choose to include only a few citations. Hence, applicant citations in the case of EPO patents may be less likely to represent ‘applicant search effort’.

In addition, how researchers compute applicant citation probability differs between USPTO and EPO patents. In the USPTO case, studies analyzing applicant citations use those provided by the applicant in the information disclosure statement (Thompson 2006; Alcácer and Gittelman 2006; Alcácer et al. 2009). In the EPO case, applicant citations are those included by the applicant in the description of the invention *and considered relevant by examiners* in their reports (Criscuolo and Verspagen 2008).

Based on these differences between USPTO and EPO, in this paper we propose a new interpretation of the probability of applicant citation: an applicant citation in an EPO examiner report indicates examiner trust in the applicant. The first objective of this study is to validate this interpretation, *ex ante* via interviews with patent examiners, applicants and other agents,^3^ and *ex post* through a quantitative test of the relation between probability of applicant citation and probability of a patent being granted at the EPO.

Our research also exposes a so far ignored dimension of the probability of applicant citations: the national characteristics of the applicant. We know that the distribution of applicant citations in patents depends on characteristics of the application procedure (patent office), of the patent (year, technology field), of the applicant (experience, organization type, co-applicants), etc. (Alcácer et al. 2009; Azagra-Caro et al. 2011; Park et al. 2017). The role of national characteristics of the applicant has not been explored, and this paper fills this gap, in consonance with our focus on examiner trust in applicants. We argue that this trust is a socially mediated construct, and that country specificities play an important role in shaping this trust, in part due to geographically-bounded cognition of examiners (Wada 2016).

Our second objective is to offer some explanations of the variation in examiner trust according to the influence of applicant country characteristics at the EPO. Based on qualitatively-informed hypotheses, we make a quantitative test of the influence of national economic and scientific strengths of applicant country, an applicant country that is a member of the European Patent Organization, and applicants and examiners that are from the same country.

## Literature review and qualitatively-informed hypotheses

### Qualifying applicant citations in EPO reports as indicators of ‘examiner trust’

EPO patent citations are classified into categories, indicated by upper case letters. Patent examiners decide which of the citations in the application text are relevant to the examination, and therefore, should be included in their reports (category D). The examiner also adds citations, usually many more than the included by the applicant, and in several categories (X, Y, A, etc.).^4^ Hence, the calculation of probability of applicant citation in EPO patents is based on citations included by the applicant in the description of the invention *which are considered relevant by examiners* in their reports (Criscuolo and Verspagen 2008; Baruffaldi and Raffo 2017). Table 1 presents the figures for the distribution in the sample studied in this paper.

**Table 1:** Citation categories in an EPO examiner report

Category	Meaning (in examiner reports established for a European patent application, or in the European Patent Register)	Distribution (*n* = 3,663,276) (%)
A	Technological background. Used for a document representing ‘state of the art not [regarded as] prejudicial to the novelty or inventive step of the claimed invention’	43.31
X	Highest possible level of relevance. ‘Applicable where a document is such that when taken alone, a claimed invention cannot be considered novel or cannot be considered to involve an inventive step’	32.71
Y	Document particularly relevant if combined with another ‘Y’ document	12.48
D	Document cited in the application by the applicant itself	6.54
P	Intermediate documents, i.e. ‘[documents] published on dates falling between the date of filing of the application being examined and the date of priority claimed, or the earliest priority if there is more than one’	3.23
E	Potentially conflicting patent documents, i.e. ‘bearing a filing or priority date earlier than the filing date of the application searched… but published later than that date and the content of which would constitute prior art relevant to novelty’	1.08
T	Documents relating to the theory or principle underlying the invention	0.30
I	This category is not used in search reports but in the European Patent Register for a single ‘X’ document ‘particularly relevant for reasons of inventive step’. See category X	0.21
L	Documents cited for other reasons. For example, if an examiner ‘considers that a publication, although undated, is highly relevant to the invention and can therefore be considered to be of interest to the applicant or third parties, he may choose to cite the publication in the search report as an ‘L’ document’	0.15
O	Non-written disclosure	0.01

*Source*: column 2 (meaning), adapted from Guidelines for Examination in the European Patent Office September 2013; column 3 (distribution), own elaboration (sum equals 100%)

To overcome the limitations of the term ‘applicant search effort’ coined by Alcácer et al. (2009) in the EPO context, we propose the alternative label ‘examiner trust in applicants’. This takes account of the fact that examiners mediate the appearance of applicant citations in their report. We asked our EPO and non-EPO examiner-informants whether they considered the term appropriate, and they had a tendency to agree, although weakly. More convincing was their spontaneous reaction to our question concerning applicant citations in the description of the invention that they dismissed for their reports: they described them as ‘*irrelevant’, ‘noisy’, ‘tall story’, ‘smoke screen’,* etc., words and terms that revolve around the concept of ‘trust’. EPO Examiner 2 put it this way:

Applicants usually try to cite infrequently in order not to reveal much, and very often what they cite does not have to do with their claimed invention –once you make the search, you don’t care what they have sent you. You have to remain skeptical.

Inventors and agents tended to agree more strongly with the label ‘trust’ to refer to acceptance of a citation by the examiner:

If an examiner considers five documents to be relevant and three come from you, this means that the criteria are coincident and you have done a good patent job. When you cite fewer documents, then you get fewer Ds.

Agent 2.

Therefore, we propose to use the term ‘examiner trust in applicants’ in the rest of this paper. In addition, to back up this idea, we argue that the probability of finding an applicant citation will be higher in granted patents. If examiner citations imply that the application is not well documented and/or not very novel, we would expect a positive link between probability of applicant citation and having the patent granted, especially at the EPO because of the quality of the examination process (de Saint-George and de la Potterie 2013). In the words of Inventor 1:

I always add a similar number of citations to my patents. I have noticed that [EPO] examiners added fewer citations to my most innovative patent.

Thus, we hypothesize:

#### Hypothesis 1

Examiner trust in applicants to the EPO is more likely to be found in granted patents.

### Examiner trust in national economic and scientific strengths

To argue about the effect of economic and scientific strengths of countries on examiner trust measured by applicant citation probability, we have to go back to the rationale behind citations in patent documents. Applicants use citations to claim the novelty of their invention, and improvements in the state of the art. Examiners use citations other than applicants’ for the opposite reason: to justify that novelty is limited, given the existence of similar inventions or bodies of knowledge. This would imply that examiner reports with many applicant citations reflect inventions that the examiner considers quite novel, because there is no need to refute many claims.

Wealthier and scientifically stronger countries are more likely to create conditions favorable to the appearance of novelty, thus inducing examiners to trust applicants. Some evidence suggests so at regional level, where richer regions present higher proportion of applicant vis-à-vis examiner citations (Azagra-Caro et al. 2011). At country level, per capita GDP and research and development (R&D) are positively associated with higher innovative capacity measured by patents (Furman et al. 2002), and higher knowledge flows proxied by backward references in patents (Azagra-Caro and Consoli 2016). Hence, we find reasons to postulate:

#### Hypothesis 2

National economic and scientific strengths favor examiner trust in applicants to the EPO.

### Examiner trust in applicants from the same country bloc

Country bloc effects may also play a role in determining examiner trust. Specifically, we are interested in whether there is a country bloc effect similar to the one shown by Alcácer et al. (2009) in the USPTO case: US applicants receive fewer examiner citation shares than non-US applicants. Nonetheless, since the EPO is an international organization, this country bloc effect needs to be reformulated.

In national patent offices, applicants seek protection in one country only, which may or may not be their own country and which allows examiners to differentiate clearly between domestic and foreign applicants. In the EPO, the distinction between domestic and foreign applicants is blurred since patents provide protection for inventions in many countries, and examiners include people of different nationalities. National patent offices may judge foreign applicants according to different criteria than those applied by the applicant’s domestic country patent office whereas the EPO applies international criteria, agreed upon by signatory countries to the European Patent Convention, i.e. members of the European Patent Organization (EPOrg). Hence, we propose that if a country bloc effect exists, it might be evident for member countries of the EPOrg. This might be due to better knowledge of the ‘rules of the game’ among signatories to the EPOrg, and might translate into lower probability of applicant citations for non-EPOrg countries. Mistakes due to the lack of a patenting culture can be very graphic, as described by EPO Examiner 5:

Sometimes I have had copies of PhD theses as patent applications: they are not well structured. The claims are wrongly formulated, with several sentences, etc. This complicates our lives because we have to raise many objections.

The rules of the game can take the form of the ‘problem–solution’ approach customary at the EPO, as pointed out by EPO Examiner 1:

Most EPO applications come from Germany, US and Japan. EPO applications of European origin, particularly those from German companies, follow the problem–solution approach more closely, i.e. they identify the state-of-the-art, then describe the problem to be solved […] and which characteristic features of the invention solve [it]. In addition, they often employ a European patent attorney which contributes to the correct drafting of the claims […]. The documents cited by the applicant in the patent application tend to be of high relevance. In contrast, US-originated applications are copies of the first application at the USPTO. Because of the US [duty of candor], US applicants may cite many irrelevant, noisy documents which we [EPO examiners] do not consider in the search report. On the other hand, many EPO applications from Japan are written by Japanese patent attorneys and translated into English. These translations are sometimes difficult to understand and a bit messier.

The predominance of German, US, and Japanese applications, the fact that US-originated patents do not increase applicant citation probability in EPO examiner reports, despite the US duty of candor, and the importance of knowing the ‘rules of the game’ is also acknowledged by EPO Examiner 2:

German agents probably represent around 20 percent and they follow the style of the German Patent Office, and EPO is the offspring of the German Patent Office […]. Without knowledge of the system, [the application process] can be disastrous because applicants may be unaware of the norms, and just because of problems related to not following the correct format, they can be rejected even in the first phase.

These extracts are illustrative of the ‘rules of the game’, which may appear in the form of a country bloc effect at the EPO equivalent to the USPTO preference for US applicants. Therefore, we hypothesize that:

#### Hypothesis 3

Examiner trust in applicants to the EPO is higher if applicants’ countries are members of the EPOrg.

### Examiner trust in applicants from the same country as the examiner

Having isolated a country bloc effect, it is possible that examiner nationality might have an effect in the case of EPOrg countries. This type of bias in patent systems has been explored from different angles. For instance, the role of nationality in the patenting process determines firms’ preferences for an application to their national office –the ‘home advantage’ effect, e.g. European firms apply disproportionately to the EPO (Criscuolo 2006). Similarly, the EPO sometimes refuses Japanese Patent Office (JPO) applications that have been granted by the USPTO, while the JPO sometimes refuses EPO applications that have been granted by the USPTO, suggesting a local bias (Palangkaraya et al. 2011). The positive effect of being a local inventor on the likelihood of being granted a patent at the EPO and JPO ratifies such bias (Webster et al. 2014).

Collins and Wyatt (1988, p. 73) detect national chauvinism in citations to non-patent literature in US genetics patents: ‘it appears that every country is its own best citer’. However, Meyer (2000) finds no signs of national chauvinism in nanotechnology patent applications to the USPTO from Swedish applicants but finds evidence of cosmopolitanism (i.e. frequent citing of foreign countries) which might be due to Sweden’s small country size. If we assume the presence of national chauvinism in examiner citation probability, it might emerge in higher citations to the same home country of the examiner and the applicant. This could be due to a common cultural background which includes knowledge of similar references (including references in the native language of both examiner and applicant).

If such biases exist at the EPO, they are not encouraged institutionally, and are more likely to be discouraged. Some sort of official position was described as follows:

Applications are searched and examined according only to their substance, and regardless of the type (individual, academic, company), nationality, language, company size or other characteristics of the applicant or inventor. This is apparent from the European Patent Convention and also is a clear requirement of Article 3 of the TRIPS Agreement under the WTO to which nearly all EPC contracting states are members.

EPO Examiner 3.

EPO examination committees are organized on the basis of technical expertise in the technology at stake. Membership of these committees or ‘divisions’ rotates, and members are appointed either by the area director or by random selection. The EPO allows examiners to be of the same nationality as the applicants:

Otherwise it would be complicated to manage, given that there are so many German examiners and applicants! However, the examination division is constituted of three members, and normally the idea is that they are from different nationalities to reinforce the neutrality of the decisions.

EPO Examiner 1.

This is an informal policy since, according to EPO Examiner 4, there are no specific directives. Thus, coincidence between examiner and applicant nationality is possible, and in some cases might be advisable:

Sometimes it is managed so that one examiner shares the applicants’ native language, just in case it is necessary in the oral round.

EPO Examiner 2.

In addition, Agent 2 (a former EPO examiner) had experienced exceptions to the rules of rotation and random:

Sometimes, yes, it is true that out of curiosity, the head of division or examiner may pick up an application originated in the same nationality as the examiner.

Despite evident organizational goodwill and best practice, it is difficult to rule out that individuals will form their own views. In our conversations with EPO informants, it was clear that they held opinions about patent applicants, agents, and citations from EPOrg member countries –sometimes negative opinions. In these cases, some informants agreed that this could translate into lower probability of applicant citations since often the inclusion of relevant documents by applicants is dependent on the national patenting culture. EPO Examiner 6 acknowledged that there is a stronger culture of patenting in the North than in the South of Europe; thus, he expected documents cited by Northern applicants to be closer to the state-of-the-art than those cited by Southern applicants. Understandably, the role played by country specificities has triggered debate outside the EPO:

EPO examiners’ nationality bias is difficult to get rid of –it is anthropological. In some of our analyses, we [the speaker and colleagues] have noticed that for some silly inventions, someone from [Country A] patents them more easily at the EPO than someone from [Country B] […]. It may be the same patent, but one looks at it with different eyes […]. It happens even to me with patents from [a less developed country]! Because of the different writing style, or because the innovative level of some places cannot match that of others. Of course it is the examiner’s role to overcome this prejudice.

Non-EPO European Examiner.

The above statement points out to a cultural bias underlying the possible national preferences of EPO examiners. Inventor 2 considered some plausible causes. His case is interesting because he was the most prolific inventor in one of the countries and period in our sample (over 250 EPO and/or PCT patents), and because he has been based for long periods in two different countries. He has also been the inventor on patents applied for by companies from these two countries, one a Central European country and one a Southern European country:

EPO examiners tend to add more citations to applicants from other nationalities. That’s because prior art has a national component based on the examiner’s memory experience. It is not a personal but an educational issue […]. An examiner [from the Central European country of the inventor] is looking for citations [from that Central European country]; a South European examiner, more for South European citations.

Inventor 2.

This suggests that culture shapes examiners’ inclusion of citations. Wada (2016) also argues about the cognitive boundary of examiners who will tend to cite more their country’s patents in technologies where their countries stand out, because of their local education. Hence, although discouraged institutionally, and perhaps unconsciously at individual level, the same sort of national bias that applies to other parts of the patenting process may apply also to citation practices (Criscuolo 2006; Palangkaraya et al. 2011; Webster et al. 2014). This leads to our final hypothesis:

#### Hypothesis 4

Examiner trust in applicants to the EPO is higher if examiners and applicants are from the same country.

### Control variables in the analysis of examiner trust in applicants

#### EPO procedures: phase and route

Citations can originate in several phases of an EPO patent’s life, not every phase generates both applicant and examiner citations and the probability of finding an applicant citation may vary according to the phase of the application procedure. Criscuolo and Verspagen (2008) analyze citations from the search report, without providing details on which search report they consider. To help clarify this issue, this section introduces an explanation of the citation phases (for a more comprehensive explanation, see Wada 2016).

For direct EPO applications, there are two relevant phases: the EPO search procedure and the substantive examination. The EPO search procedure ends with the publication of an EPO search report, which documents whether the application fulfils the requirements of novelty and non-obviousness. It is not binding but it signals to the applicant the likelihood of the patent being granted, informing the decision about whether to pay the extra fees required to continue the process.

During the substantive examination, examiners assess industrial applicability in addition to novelty and non-obviousness. This phase ends with the denial of the application or with the publication of the granted patent. During the examination, both the examiner and the applicant may include additional relevant citations.

Moreover, the EPO allows indirect applications through the Patent Cooperation Treaty (PCT) procedure. The PCT authorizes some patent offices to be International Search Authorities and run a unified application protocol, valid in all signatory countries. In this case, an additional phase produces applicant and examiner citations, prior to the first two phases described above, and it results in an international search report published by the PCT. After publication of the international search report, the applicant can designate the EPO as ‘region’ of protection. Then, the application enters the ‘European regional phase’ and becomes a Euro-PCT application, which enters the two phases described above.

#### Citation characteristics: science base

Citations can be to patent literature or to non-patent literature. The pioneering research of differences between examiner and applicant citations focuses on patent literature (Thompson 2006; Alcácer and Gittelman 2006; Criscuolo and Verspagen 2008). However, citations to non-patent literature are interesting because they signal science relatedness (Callaert et al. 2014) and their study is mandatory in the context of the EPO because they are more frequent there than in other patent offices (Michel and Bettels 2001; Callaert et al. 2006). By controlling for this variable, we test whether applicants are more likely than examiners to cite non-patent literature, extrapolating from US evidence that examiners rarely cite non-patent literature (Sampat 2004).

#### Patent characteristics: year and technology class

Other patent characteristics also matter for probability of applicant citation, e.g. time and technology class. The evolution of EPO applicant citation probability has been declining for the period 1985–2000 (Criscuolo and Verspagen 2008). Other works have documented a prominent number of applicant citations in chemical technologies (Alcácer et al. 2009; Azagra-Caro et al. 2011; Park et al. 2017).

#### Applicant characteristics: organization type and experience

The expected effect of type of organization on examiner citation probability is tricky. Firms are the main patentees and may be more familiar with the rules of the game, which in turn may make their applications less subject to insertion of examiner citations. However, scientific institutions, i.e. universities and government labs are closer to the science base and also are often familiar with the relevant literature, which may reduce examiner added citations. If we accept that scientific institutions dominate a second body of literature in addition to that of firms, we may predict higher applicant citation probability. There are other reasons to expect so: serial academic invention is based on the quality of earlier patents (Lawson and Sterzi 2014), and this reputation may pay off in the form of lower examiner citation probability. Then there is the case of patents co-applied for by scientific and non-scientific institutions, which evidence technological cooperation between the partners. This interaction has been shown to have potential benefits for firms, e.g. practical solutions to technical needs, even in low-tech regions (Ortega-Colomer 2013) or access to doctoral graduates (Garcia-Quevedo et al. 2012). It also has benefits for academics, such as increasing research output (Villanueva-Felez et al. 2013). If university and/or government-industry interaction are so useful, one would expect to find them linked to higher applicant citation probability, because they would originate better documented and more novel patent applications.

Regarding applicant experience, the number of applicant citations decreases with the number of an applicant’s filings in the USPTO case (Alcácer et al. 2009): large applicants prefer ‘broad patent portfolios, with relatively low value placed on any single invention’ (p. 426). An alternative reason may be that applicants include unrelated cites after the invention or omit relevant cites for strategic reasons (Breschi and Lissoni 2005). Perhaps experienced applicants learn how to ‘cheat’ the system, and hide a higher number of relevant references. One of our interviewees also illustrated this point:

Multinationals make noise: they do research, have patent departments, fill applications for everything they do (no matter whether it is good or not) and they get patents if they can, regardless of their repercussion. There is a huge number of patents that are not going to yield anything, but they threaten competitors […], allow the companies to say, ‘Here I am’, and then they withdraw them.

EPO Examiner 2.

### Model, data and variables

In order to verify hypotheses 1–3, we estimate the following model:

1 $$\begin{aligned} & \Pr \left( {appcit_{ijklt} } \right) = f\left( {grant_{j} ,\;per\;capita\;GDP_{lt - 2} ,\;GERD\;intensity_{lt - 2} ,\;EPOrg\;member_{lt} ,\;X_{i} ,X_{j} ,X_{k} } \right) + \varepsilon_{i} + \theta_{l} \\ \end{aligned}$$

where examiner trust is modelled as the probability of applicant citation, *appcit*, equal to 1 if the citation is inserted by the applicant and 0 if it is inserted by the examiner. This probability varies according to the characteristics of the citation *i*, the patent *j*, the applicant *k,* and the applicant country *l*. Our target independent variables are whether the patent is a *grant*, national economic and R&D characteristics (measured by *per capita GDP* and gross expenditure on research and development over GDP, i.e. *GERD intensity*) and whether the applicant country belongs to the EPOrg (*EPOrg member*). The year of the patent application *t* is lagged two periods for national economic and R&D characteristics to prevent endogeneity biases. Finally, ϵ is the idiosyncratic error and θ is an unobserved cluster-effect capturing the influence of the country.

In addition, to verify hypothesis 4, which applies to EPOrg member countries, we estimate:

2 $$\begin{aligned} & \Pr \left( {appcit_{ijklt} } \right) = f\left( {grant_{j} ,\;per\;capita\;GDP_{lt - 2} ,\;GERD\;intensity_{lt - 2} ,\;EPOrg\;exam\;same\;country_{lt} ,\;X_{i} ,X_{j} ,X_{k} } \right) + \varepsilon_{i} + \theta_{l} \\ \end{aligned}$$

where *EPOrg exam same country* is the probability of finding an examiner from the same country of the applicant.

Data on patents and citations come from Patstat (October 2012 edition). We selected citations from patents where the publication authority was the EPO,^5^ from application years 1997 to 2007.^6^ Patstat classifies citations into origin types, i.e. the moment in the examination process when the citation was inserted. There are ten types of origins (coded 0-9). The only ones relevant for this study are those with a citation category, indicating that either the patent applicant or an examiner could have inserted the citation. These are citations with origins coded 0 (citations introduced during search), 2 (citations introduced during examination) and 5 (citations from the International Search Report). They represent most (82%) of the citations. Our starting count was 6.7 million citations. After removing those with missing information for citation category, patent technology class (represented by the International Patent Classification IPC) and other variables, we were left with 3.8 million citations.^7^

In the estimations, we duplicate those citations from patents with more than one applicant country, that is to say, with several applicants that do not come from the same country. We deal with this econometrically by weighting the observations by the inverse number of applicant countries. The rate of duplication (1.07) is not high, though, so this correction does not affect the results. Besides, we match Patstat to other databases on national characteristics that do not have full information for all countries and years. The final sample includes 3.7 million observations.

Patstat allows to differentiate applicant citations through the classification of citations with origins 0, 2 and 5 into several categories (coded with single letters, A, X, Y, etc.). Categories refer to the relevance of prior art to invalidate claims of novelty. Criscuolo and Verspagen (2008) call category D ‘applicant citations’ and sum the other categories as ‘examiner citations’. We follow this method. The proportion of D-citations in the total is 7% on average.

The dependent variable is a dummy that takes the value 1 if the citation comes from the applicant. A logit model with clustered robust standard errors is appropriate for this kind of data. In theory, at the citation level this variable expresses a probability. When observed at aggregate level, i.e. per year or per country as in the following figures, it expresses a percentage.

**Fig. 1:**
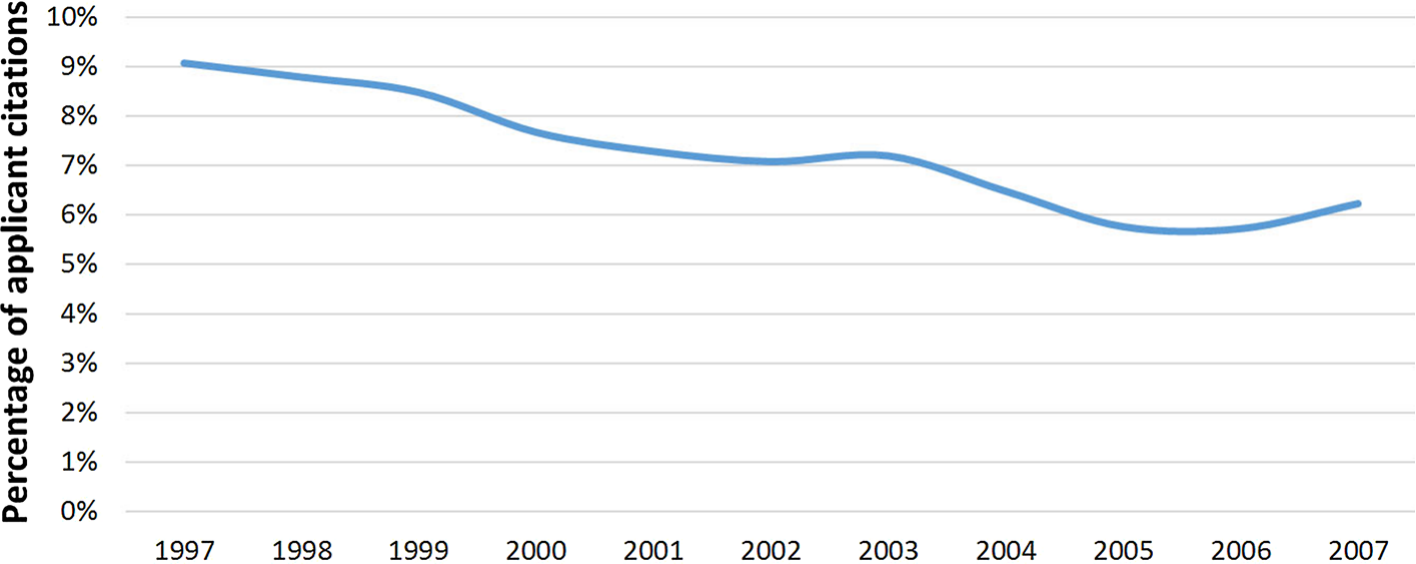
Steady decline of examiner trust at the EPO, measured as percentage of applicant citations over total (applicant + examiner citations). *N* = 3,663,276. Weight: share of number of applicant countries

The evolution of EPO examiner trust measured via percentage of applicant citations has been declining in the period of observation, 1997–2007 (Fig. 1), following the pattern detected by Criscuolo and Verspagen (2008: Fig. 1) for the period 1985–2000.

**Fig. 2:**
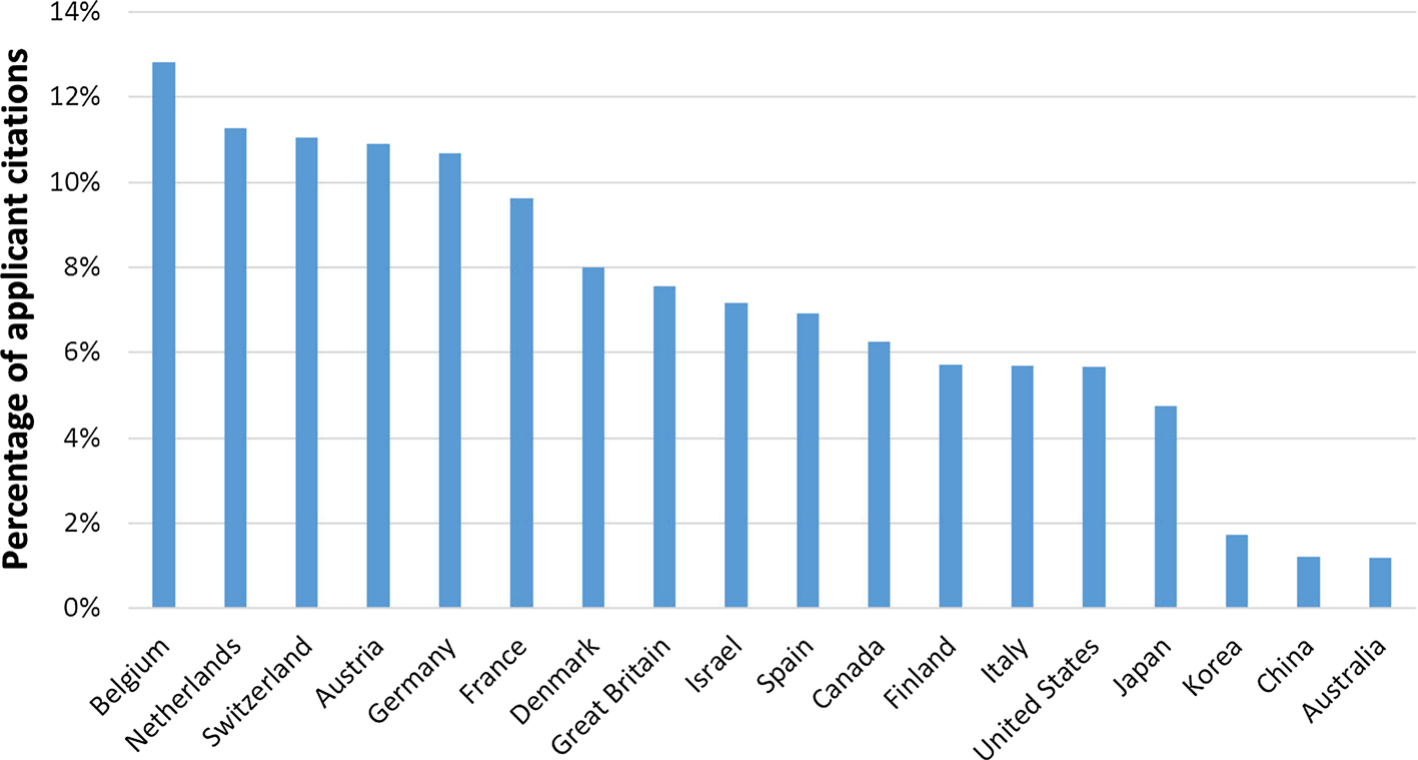
Large national variation in examiner trust at the EPO, measured as percentage of applicant citations over total (applicant + examiner citations). *N* = 3,663,276. Countries with at least 1% of EPO applications. Weight: share of number of applicant countries

National variation in the sample is clear (Fig. 2), with many core European Union states showing the highest proportion of applicant citations. A country bloc effect is already apparent since the highest examiner citation probability corresponds to countries that do not belong to the EPOrg: US, Japan, Korea, China and Australia.^8^

**Fig. 3:**
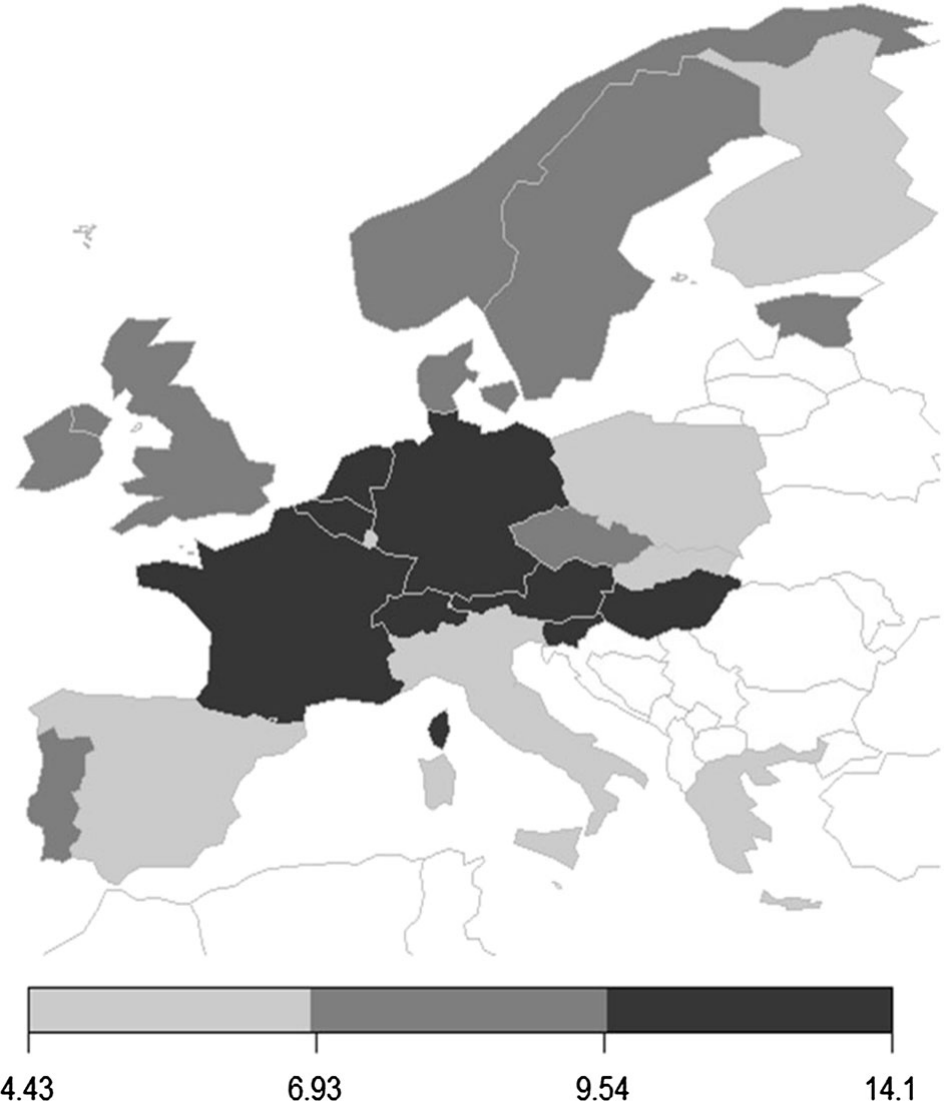
The core-periphery structure of applicant citation probability in Europe. *N* = 3,663,276. Probability calculated by the country of the patent applicant on a citation basis. Weight: share of number of applicant countries

In Europe, a pattern favoring the core European countries emerges (Fig. 3). Percentage of applicant citations is larger for Belgium, Netherlands, Switzerland, Austria, Germany and France while examiner citation probability is larger for peripheral countries such as Spain, Poland, Italy and Greece. This suggests that large applicant citation probability is related to economic wealth. There are exceptions for both sides: East European, lower income countries such as Slovenia and Hungary stand out for percentage of applicant citations and higher income countries such as Finland stand out for percentage of examiner citations. Hence, factors other than economic wealth matter, and are accounted for in the econometric estimations.

Table 2 provides information on the econometric model variables. As they appear in the next section, we will give explanations about their construction and meaning.

**Table 2:** Variable definitions and descriptive statistics (*n* = 3,663,276)

Role	Source	Level	Variables	Description	Mean	Std. Dev.	Min	Max
Dep. var.	Patstat	Citation	Applicant citation	1 if citation category is D	0.07	0.26	0.00	1.00
Controls	Patstat	Patent	Direct EPO	1 if direct EPO, 0 if EPO-PCT	0.63	0.48	0.00	1.00
		Citation	International search report	1 according to phase of origin	0.16	0.37	0.00	1.00
			European search report		0.84	0.37	0.00	1.00
			Examination report		0.00	0.06	0.00	1.00
		Patent	Filing year	Application year	2001.95	3.02	1997	2007
			A Human Necessities	Share of IPC sections	0.16	0.32	0.00	1.00
			B Performing Operations; Transporting		0.18	0.34	0.00	1.00
			C Chemistry; Metallurgy		0.14	0.29	0.00	1.00
			D Textiles; Paper		0.01	0.11	0.00	1.00
			E Fixed Constructions		0.03	0.16	0.00	1.00
			F Mechanical Engineering; Lighting; Heating; Weapons; Blasting		0.10	0.27	0.00	1.00
			G Physics		0.18	0.34	0.00	1.00
			H Electricity		0.19	0.36	0.00	1.00
		Citation	Non-patent literature	1 if non-patent literature	0.19	0.39	0.00	1.00
	ECOOM^a^	Applicant	University/Government	1 if institutional sector contains at least one university, government lab or hospital	0.05	0.22	0.00	1.00
			Nr applications	Number of applications (millions)	0.00	0.00	0.00	0.15
Testing H1	Patstat	Patent	Grant	1 if granted, 0 otherwise	0.46	0.50	0.00	1.00
Testing H2	OECD R&D Statistics	Country	Per capita GDP	GDP: Euro per inhabitant (thousands)	31.1	5.44	2.65	68.21
			GERD intensity	Total Gross R&D expenditure: Millions of PPS at 2000 prices	2.50	0.47	0.28	4.58
Testing H3	EPO Annual Reports	Country	EPOrg member	EPO member (yes/no)	0.46	0.50	0.00	1.00
Testing H4			EPO exam same country	Probability of examiner from same nationality	0.10	0.10	0.00	0.26

^a^Methodology explained in DuPlessis et al. (2009), Magerman et al. (2009) and Peeters et al. (2009)

Table 3 offers the distribution of patents in the sample, for descriptive purposes.

**Table 3:** Patent set properties (*n* = 724,254)

Variable	Number of patents	%	Variable	Number of patents	%
Direct EPO	464,046	64			
Euro-PCT	260,208	36	A Human Necessities	139,966	14
			B Perf. Op.; Transp.	188,522	19
Grants	347,633	48	C Chemistry; Metallurgy	146,193	15
Applications	376,621	52	D Textiles; Paper	16,144	2
			E Fixed Constructions	28,121	3
Filing year			F Mechanical Eng., etc.	93,769	9
1997	57,047	8	G Physics	195,061	19
1998	65,300	9	H Electricity	195,894	20
1999	64,310	9			
2000	69,948	10	University/Government	31,937	4
2001	73,367	10	Other sectors	698,115	96
2002	73,095	10			
2003	73,127	10	Germany	189,665	26
2004	68,616	9	United States of America	180,020	25
2005	65,048	9	Japan	174,155	24
2006	61,574	9	Other countries	180,414	25
2007	52,822	7			

Full counting of patents in the breakdown by IPC section

## Results

Table 4 presents the estimations of the model. The estimates are expressed as odds ratios instead of coefficients to measure effect sizes, which is particularly convenient in the case of large samples because statistically significant coefficients may hide effect sizes with no practical significance (Lin et al. 2013). An odds ratio smaller than one expresses a negative effect of a variable (i.e. the variable decreases the probability of a citation coming from the applicant).

**Table 4:** Logistic regression of the probability of examiner trust (an applicant originating a citation rather than the examiner) at the EPO (odds ratios)

	1 All applications	2 Euro-PCT	3 Direct EPO	4 All applications
Direct EPO^a^	1.14			1.31
	(0.19)			(0.19)
European search report		0.68**		
		(0.11)		
Examination report		0.14***	0.08***	
		(0.02)	(0.02)	
Grant	1.38***	1.22***	1.37***	1.36***
	(0.02)	(0.03)	(0.04)	(0.02)
Filing year	0.96**	0.96***	0.97	0.96**
	(0.02)	(0.02)	(0.02)	(0.02)
B Perf. Op.; Transp.	0.94	0.89***	0.94	0.94
	(0.04)	(0.03)	(0.06)	(0.04)
C Chemistry; Metallurgy	1.77***	1.72***	1.73***	1.77***
	(0.12)	(0.06)	(0.14)	(0.11)
D Textiles; Paper	1.58***	1.58***	1.55***	1.58***
	(0.07)	(0.08)	(0.11)	(0.07)
E Fixed Constructions	0.85**	0.78***	0.90	0.86**
	(0.06)	(0.05)	(0.07)	(0.06)
F Mechanical Eng., etc.	0.79***	0.76***	0.77***	0.79***
	(0.05)	(0.02)	(0.06)	(0.05)
G Physics	0.75***	0.75***	0.71***	0.74***
	(0.04)	(0.03)	(0.05)	(0.04)
H Electricity	0.62***	0.58***	0.60***	0.61***
	(0.06)	(0.03)	(0.07)	(0.06)
Non-patent literature	1.12	0.90	1.29	1.11
	(0.35)	(0.14)	(0.51)	(0.35)
University/Government	1.07	1.16***	1.13	1.08
	(0.09)	(0.05)	(0.15)	(0.09)
Nr applications	0.01	0.01**	0.00***	0.00
	(0.02)	(0.02)	(0.00)	(0.01)
Per capita GDP	1.02***	1.02**	1.03***	1.02***
	(0.01)	(0.01)	(0.01)	(0.01)
GERD intensity^a^	1.17*	1.08	1.21**	1.16
	(0.11)	(0.13)	(0.10)	(0.11)
GERD int. × Direct EPO				0.60***
				(0.09)
EPOrg member	2.35***	2.50***	2.00***	2.42***
	(0.22)	(0.54)	(0.18)	(0.21)
Observations	3,663,276	1,341,052	2,322,224	3,663,276
Log likelihood	− 841,944	− 285,779	− 552,093	− 840,767
Prob > *χ*^2^	0.000	0.000	0.000	0.000

*p < 0.1; **p < 0.05; ***p < 0.01

^a^Centered in models with interaction term. Clustered robust standard errors in parenthesis. No collinearity according to Variance Inflation Factors. Weight: share of number of applicant countries

Column 1 includes the specification of Eq. 1 for the whole sample of citations. Because taking into account the phases of origin requires splitting between citations from Euro-PCT and direct EPO patents, we present such a breakdown in Columns 2 and 3, respectively.

### Control variables

The dataset indicates whether the application followed a Euro-PCT or a direct EPO procedure. In Column 1, the odds ratio of ‘Direct EPO’ is not significant, showing that this procedure does not lead to higher numbers of examiner citations than the Euro-PCT procedure.

We follow with EPO procedural aspects, where we expect a strong ‘early intervention’ effect: in later phases of the application procedure, applicant citations should appear more rarely than examiner ones. Citations are coded to indicate whether the origin is the international search, the European search or the examination phase for Euro-PCT patents, or one of the last two for direct EPO patents. In Column 2, the odds ratio of ‘European search report’ is smaller than one and significant, implying that citations in this second phase of a Euro-PCT application are more likely to be associated with examiners than in the first phase. The odds ratio of ‘Examination’ is also smaller than one and significant, and smaller than the odds ratio of ‘European search report’, meaning that citations in this last phase are most likely to come from examiners than in any other phase. In Column 3, we see a similar negative effect of ‘Examination’ compared to ‘European search report’ for direct EPO applications.

The odds ratio below 1 of the trend variable ‘filing year’ captures the decline of applicant citation probability shown in Fig. 1. This decline is attributable to Euro-PCT applications rather than to direct EPO patents (Columns 2–3).

For IPC sections, the reference category is ‘A Human Necessities’ (we applied fractional counts, so the IPC variables are shares of total number of sections in a given patent). Some technologies exhibit larger applicant citation probability: ‘C Chemistry; Metallurgy’ and ‘D Textiles; Paper’. The rest have larger examiner citation probability (sections B, E, F, G and H), although sometimes only significantly for Euro-PCT applications (sections B and E).

We test whether applicants are more likely than examiners to cite non-patent literature. The non-significant sign of ‘Non-patent literature’ shows that this is not the case.

The dummy for scientific institutional affiliation of the applicant (university/government) takes company and individuals as the benchmark. Co-applications with scientific institutions, a small portion of the total, are included in the dummy. Its non-significant odds ratio indicates that scientific institutions receive the same share of examiner citations than other institutions. However, the odds ratio becomes larger than one and significant for Euro-PCT patents, suggesting that the more international the patenting process is, the higher the credit examiners give to scientific applicants. The reason may be that public research organizations must be very selective about the patents they apply for through such a costly procedure like the PCT, and as a result they file only inventions which examiners will distinguish as outstanding.

The probability of applicant citations decreases with the increase in the number of an applicant’s filings. The effect is not significant on the aggregate, but it is for each subsample (Euro-PCT and direct EPO), which suggests the presence of non-linearities. The present evidence shows that the EPO and the USPTO are similar in this regard (see Alcácer et al. 2009).

### Grants

The sample comprises applications and grants. Patstat includes codes that allow identification of whether an application was granted or not. This is controlled for in the models by the dummy variable ‘Grant’. The estimated odds ratio is larger than one and significant: examiner trust is 20–40% more likely to be found in granted patents. Hence, we can confirm a positive link between probability of applicant citations and having the patent granted in the EPO case, unlike the USPTO case. This is evidence in favor of Hypothesis 1, and it is robust across specifications from here onwards.

### Country characteristics

The variables per capita GDP and GERD intensity present larger than one and significant odds ratios. They provide evidence that those countries with a favorable endowment benefit from higher examiner trust, supporting Hypothesis 2. Applicant citation probability is higher in patents from national contexts where economic wealth and R&D effort are larger. Concerning R&D, this effect is larger for direct EPO, and not significant for Euro-PCT. That is to say, the less international the patenting process is, the more credit examiners give to the scientific strength of the country. This suggests that the influence of GERD intensity is moderated by patenting route. We test this in column 4, where we pool the sample like in column 1, and add an interaction term between GERD intensity and direct EPO application. The interaction enters with a larger than one, significant sign and confirms the moderating role of following the direct EPO procedure on the effect of GERD intensity. Actually, it absorbs the significance of the main term: the direct effect of GERD intensity becomes not significant. The EPO is closer to the country level than the PCT, which may explain why the EPO is more sensitive to country characteristics.

In the model, a dummy equal to 1 if the applicant country belongs to EPOrg captures the influence of this membership. The estimate, larger than one and significant, verifies that there is a lower propensity for EPOrg member states to receive cites from the examiner. This is, actually, the variable with the strongest effect on the probability of a citation coming from the applicant: if the applicant country belongs to the EPOrg, the probability of applicant citation doubles. Hence, the EPO is similar to the USPTO in that outsiders are less warmly received. This is evidence in favor of Hypothesis 3.

To analyze the effect of nationality of EPO examiners within EPOrg countries, we split the sample in Table 5. Column 1 contains estimations for EPOrg countries, Column 2 for non-EPOrg ones. To build the variable ‘EPO examiner from the same country’, unfortunately, we face that EPO does not provide information about actual individuals conducting the examination, so we do not know the exact match between nationality of applicant and examiner. This is an important limitation that allows for a tentative test of Hypothesis 4. We proxy this measure by the probability of an application being examined by an examiner from the same country as the patent applicant, through information published in the EPO annual reports, which contain tables with numbers of staff in post by grade and nationality. We selected staff from Grade A only, because it includes examiners. This grade also includes legal staff and translators, which recalls that the variable is just a proxy. Nonetheless, it does exclude executive, managerial, service and other staff (Grades B and C). Numbers for EPOrg members are detailed. Numbers for non-EPOrg members are not detailed but aggregated into ‘Others’. The numbers of ‘Others’ are small, so we decided not to use it. For a given country and year, its share over the total Grade A staff defines our variable.

**Table 5:** Logistic regression of the probability of examiner trust (an applicant originating a citation rather than the examiner) at the EPO: EPOrg versus non-EPOrg (odds ratios)

	1 EPOrg	2 Non-EPOrg
Direct EPO^a^	0.95	1.41***
	(0.05)	(0.17)
Grant	1.34***	1.29***
	(0.02)	(0.04)
Filing year	0.97***	0.96
	(0.00)	(0.05)
B Performing Operations; Transporting	0.89*	0.98
	(0.06)	(0.04)
C Chemistry; Metallurgy	1.80***	1.65***
	(0.11)	(0.13)
D Textiles; Paper	1.53***	1.57***
	(0.07)	(0.18)
E Fixed Constructions	0.85***	0.83
	(0.05)	(0.12)
F Mechanical Engineering; etc.	0.74***	0.80*
	(0.05)	(0.10)
G Physics	0.76***	0.68***
	(0.05)	(0.03)
H Electricity	0.66***	0.54***
	(0.05)	(0.06)
Non-patent literature	0.73***	1.67
	(0.04)	(0.75)
University/Government	1.20**	1.00
	(0.10)	(0.11)
Nr applications	0.00***	0.01
	(0.00)	(0.06)
Per capita GDP	1.02**	1.03**
	(0.01)	(0.01)
GERD intensity^a^	1.04	1.13
	(0.09)	(0.24)
GERD intensity × Direct EPO	0.76***	0.79
	(0.06)	(0.26)
EPO exam same country	2.17**	
	(0.72)	
Constant	1,668,147	1,995,129
	− 480,079	− 356,528
Observations	0.000	
Log likelihood	1.05	0.59***
Prob > *χ*^2^	(0.04)	(0.07)

*p < 0.1; **p < 0.05; ***p < 0.01

^a^Centered in models with interaction term. Clustered robust standard errors in parenthesis. No collinearity according to Variance Inflation Factors. Weight: share of number of applicant countries

In Table 5, the larger than one and significant odds ratio of the probability of an application being examined by an examiner from the same country as the applicant provides support for the national bias assumption. Patents from countries where more examiners come from tend to have more applicant citations. That is to say, examiners are more likely to add fewer citations to patents from applicants of the same nationality as themselves. Hence, the evidence supports Hypothesis 4.

The breakdown by country bloc offers some other insights in terms of differences between both groups. First of all, the neutral effect of direct EPO filings in Table 4 is the consequence of aggregating a negative, significant effect for EPOrg countries and a larger than one, significant effect for non-EPOrg countries. The decline of examiner trust and the negative effect of applicant experience are attributable to EPOrg countries. Regarding science relatedness, we can see that the country bloc context also matters: non-EPOrg countries benefit from a higher scientific knowledge base, as measured through non-patent literature, whereas EPOrg countries benefit from having scientific applicants. A possible interpretation is that, as outsiders, non-EPOrg applicants have to demonstrate their science base in smaller pieces of knowledge, while insiders (EPOrg applicants) just need to show their affiliation.

Finally, and regarding one of our variables of interest, it is worth noticing that the interaction term ‘GERD intensity × Direct EPO’ is significant only within EPOrg. This implies that Hypothesis 2 is valid regarding the positive effect of national scientific strength only for EPOrg countries, and moderated through the direct EPO route for patenting. It also contributes to ratify that closeness between patent office and the country level favors the positive effect of national scientific strengths on examiner trust: not only this is effect is more important in EPO than in PCT, but it is also more important for EPOrg countries than for non-EPOrg countries within EPO (Table 5).

### Robustness checks

In this section, we test whether our results are resistant to changes in the sample based on three criteria: belonging to the three top patenting countries, inventor nationality, use of a weighted sample according to application type, and randomly reduced subsamples.

Our interviewees referred often to Germany, Jadpan, and the United States as the three most frequent patenting countries at the EPO. Our data confirm that these three countries account for 73% of all EPO patents, which might affect our hypotheses. To explore this possibility, Table 6 reports separate regressions for the top patenting countries versus the rest. All the models confirm that examiner trust is more likely to be found in granted patents and that national economic strengths enhance examiner trust in applicants. In the case of scientific strengths, except for countries other than the top three, this applies also if non-EPOrg members are included (Model 2). Models 1 and 2 confirm that examiner trust is increased if the applicant country is a member of EPOrg. Model 1 confirms this for Germany versus Japan and the US, and Model 2 confirms it for the remaining countries. Model 3 confirms that if only EPOrg countries with the exception of Germany are included, country coincidence of examiner and applicant fosters examiner trust in the applicant.^9^

**Table 6:** Logistic regression of the probability of examiner trust (an applicant originating a citation rather than the examiner) at the EPO: three largest patenting countries versus rest of countries (odds ratios)

	1 3 largest	2 Other	3 Other (EPOrg only)
Direct EPO^a^	0.85	0.96	0.98
	(0.13)	(0.07)	(0.05)
Grant	1.33***	1.38***	1.34***
	(0.03)	(0.05)	(0.05)
Filing year	0.96	0.95***	0.97***
	(0.04)	(0.02)	(0.01)
B Perf. Op.; Transp.	1.00	0.80***	0.79***
	(0.04)	(0.05)	(0.05)
C Chemistry; Metallurgy	1.82***	1.66***	1.67***
	(0.13)	(0.12)	(0.13)
D Textiles; Paper	1.70***	1.35***	1.48***
	(0.08)	(0.13)	(0.11)
E Fixed Constructions	0.92	0.77***	0.79***
	(0.09)	(0.07)	(0.07)
F Mechanical Eng.; etc.	0.84**	0.65***	0.65***
	(0.07)	(0.03)	(0.03)
G Physics	0.75***	0.71***	0.72**
	(0.05)	(0.08)	(0.09)
H Electricity	0.65***	0.53***	0.55***
	(0.09)	(0.03)	(0.03)
Non-patent literature	1.20	0.88***	0.83***
	(0.60)	(0.04)	(0.02)
University/Government	0.95	1.39***	1.32***
	(0.12)	(0.08)	(0.08)
Nr applications	0.00***	659.56	37,100.82
	(0.00)	(11,215.49)	(500,195.76)
Per capita GDP	1.03**	1.02*	1.02**
	(0.01)	(0.01)	(0.01)
GERD intensity^a^	1.21	1.13	1.06
	(0.36)	(0.12)	(0.09)
GERD intensity × Direct EPO	0.35***	0.85	0.72***
	(0.02)	(0.10)	(0.04)
EPOrg member	2.61***	2.38***	
	(0.41)	(0.73)	
EPO exam same country			3.88***
			(1.80)
Observations	2,678,471	984,805	712,447
Log likelihood	− 619,971	− 219,566	− 182,579
Prob > *χ*^2^	0.000	0.000	0.000

*p < 0.1; **p < 0.05; ***p < 0.01

^a^Centered in models with inteaction term. Clustered robust standard errors in parenthesis. No collinearity according to Variance Inflation Factors. Weight: share of number of applicant countries

The country classification of patents has so far been based on the applicant’s country. We did not consider the inventor’s country. D citations originate in the inventor, or the applicant, or the person responsible for filing the patent application, e.g. the firm’s intellectual property rights manager, or an external patent attorney. This does not allow us to identify who inserted a citation but the applicants have the ultimate responsibility for the inclusion of citations, so focusing on applicant nationality seems appropriate. In addition, according to our patent inventor and agent informants, in large companies, inventors do not prepare the patent applications, so very few of the citations included originate in them; patent attorneys tend to be of the same nationality as the applicant but not necessarily the inventor. Finally, many examiners informed us of the importance of the patent applicant compared to the inventor in relation to search of the prior art, as exemplified by EPO Examiner 1:

At the USPTO it is compulsory that all inventors are mentioned in the application […]. In Europe, inventors are mentioned but the legislation is not so strict as in the USA. In general, the information on the inventors is not relevant for us [EPO examiners]. Companies are responsible for the application. The bulk of patents comes from multinational companies which set the standards –not the inventor. Mother companies pick up ideas among all those presented by employees even from foreign subsidiaries. Because of their local patent agents or centralized patent department, they may have their own referencing style, e.g. some of them will be more concise or know better what they want.

Nevertheless, it is relatively straightforward to test whether the inventor country matters. We identified patents on which applicant and inventor country were different, and found them to be a minority (16%). Table 7 presents the regression results for the remaining sample (84%).

**Table 7:** Logistic regression of the probability of examiner trust (an applicant originating a citation rather than the examiner) at the EPO: coincidence of applicant and inventor country sample (odds ratios)

	1 Euro-PCT	2 Direct EPO	3 POrg	4 Non-EPOrg
Direct EPO^a^			1.05	0.59***
			(0.04)	(0.07)
European search report	0.76			
	(0.16)			
Examination report	0.16***	0.08***		
	(0.03)	(0.02)		
Grant	1.21***	1.38***	1.34***	1.28***
	(0.03)	(0.05)	(0.02)	(0.05)
Filing year	0.96***	0.97	0.97***	0.96
	(0.02)	(0.02)	(0.00)	(0.05)
B Perf. Op.; Transp.	0.90***	0.96	0.90	1.01
	(0.02)	(0.06)	(0.07)	(0.04)
C Chemistry; Metallurgy	1.74***	1.75***	1.83***	1.66***
	(0.07)	(0.15)	(0.11)	(0.12)
D Textiles; Paper	1.62***	1.62***	1.59***	1.60***
	(0.10)	(0.12)	(0.08)	(0.20)
E Fixed Constructions	0.79***	0.92	0.86***	0.88
	(0.04)	(0.07)	(0.04)	(0.11)
F Mechanical Eng.; etc.	0.77***	0.80***	0.76***	0.82
	(0.02)	(0.07)	(0.05)	(0.10)
G Physics	0.76***	0.73***	0.78***	0.70***
	(0.02)	(0.05)	(0.05)	(0.03)
H Electricity	0.60***	0.61***	0.68***	0.55***
	(0.03)	(0.07)	(0.05)	(0.07)
Non-patent literature	0.92	1.32	0.72***	1.73
	(0.15)	(0.56)	(0.05)	(0.80)
University/Government	1.15***	1.13	1.22**	0.99
	(0.05)	(0.16)	(0.11)	(0.12)
Nr applications	0.01**	0.00***	0.00***	0.00
	(0.03)	(0.00)	(0.00)	(0.00)
Per capita GDP	1.02**	1.03***	1.03**	1.03**
	(0.01)	(0.01)	(0.01)	(0.01)
GERD intensity^a^	1.07	1.25**	1.03	1.17
	(0.14)	(0.11)	(0.10)	(0.26)
GERD intensity × Direct EPO			0.74***	0.78
			(0.07)	(0.26)
EPOrg member	2.82***	2.08***		
	(0.73)	(0.19)		
EPO exam same country			2.26**	
			(0.81)	
Observations	1,110,523	1,977,947	1,339,130	1,749,340
Log likelihood	− 253,635	− 504,105	− 428,692	− 327,371
Prob > χ2	0.000	0.000	0.000	0.000

*p < 0.1; **p < 0.05; ***p < 0.01

^a^Centered in models with inteaction term. Clustered robust standard errors in parenthesis. No collinearity according to Variance Inflation Factors. Weight: share of number of applicant countries

Our hypotheses and other results are supported in every case: examiner trust in applicants is positively associated to granted patents and increases with national economic and scientific strength, examiner coincidence with country bloc and applicant nationality.

Now, as we saw in section ‘Model, data and variables’, we lose many observations because of missing citation information. The main reason is that Euro-PCT and recent patents tend to include less information, probably because of delays in data gathering by Patstat. That is to say, our previous sample contained fewer Euro-PCT and recent applications than the original population. To correct this possible bias, we produced a stratified sample where the ratio of patents by procedure (Euro-PCT or direct EPO) and year (1997–2007) was non-significantly different from the original database of 6.7 million observations. Specifically, the contingency tables of procedure and year show the same distribution in the original database and in the stratified sample. The new sample size is around 800,000 observations. The subgroup that limited the sample size was the Euro-PCT applications in the last year, 2007. This is coherent with the fact that we found less patents in population with data for later years and Euro-PCT patents. The results, in Table 8, replicate the previous estimations.

**Table 8:** Logistic regression of the probability of examiner trust (an applicant originating a citation rather than the examiner) at the EPO: stratified sample (odds ratios)

	1 Euro-PCT	2 Direct EPO	3 EPOrg	4 Non-EPOrg
Direct EPO^a^			0.95	1.39***
			(0.03)	(0.16)
European search report	0.80*			
	(0.10)			
Examination report	0.17***	0.14***		
	(0.03)	(0.05)		
Grant	1.24***	1.40***	1.31***	1.30***
	(0.04)	(0.04)	(0.03)	(0.06)
Filing year	0.95***	0.97	0.97***	0.95
	(0.02)	(0.02)	(0.01)	(0.04)
B Perf. Op.; Transp.	0.92***	0.97	0.90	1.02
	(0.03)	(0.08)	(0.06)	(0.04)
C Chemistry; Metallurgy	1.73***	1.77***	1.79***	1.69***
	(0.07)	(0.18)	(0.09)	(0.14)
D Textiles; Paper	1.63***	1.69***	1.60***	1.73***
	(0.09)	(0.17)	(0.07)	(0.23)
E Fixed Constructions	0.83***	0.94	0.85***	0.89
	(0.04)	(0.08)	(0.05)	(0.08)
F Mechanical Eng.; etc.	0.82***	0.83*	0.78***	0.87
	(0.03)	(0.08)	(0.04)	(0.10)
G Physics	0.77***	0.72***	0.78***	0.71***
	(0.03)	(0.06)	(0.05)	(0.02)
H Electricity	0.60***	0.62***	0.64***	0.58***
	(0.03)	(0.06)	(0.05)	(0.05)
Non-patent literature	0.92	1.37	0.72***	1.57
	(0.14)	(0.58)	(0.04)	(0.55)
University/Government	1.21***	1.15	1.16***	1.12
	(0.05)	(0.12)	(0.06)	(0.09)
Nr applications	0.00**	0.00***	0.00***	11.73
	(0.01)	(0.00)	(0.00)	(66.39)
Per capita GDP	1.02**	1.02***	1.02*	1.02**
	(0.01)	(0.01)	(0.01)	(0.01)
GERD intensity^a^	1.09	1.25***	1.06	1.26
	(0.12)	(0.09)	(0.08)	(0.24)
GERD intensity × Direct EPO			0.75***	0.78
			(0.06)	(0.22)
EPOrg member	2.91***	2.03***		
	(0.49)	(0.17)		
EPO exam same country			2.05**	
			(0.74)	
Constant	519,899	306,447	351,434	474,912
	− 105,881	− 71,871	− 102,298	− 75,219
Observations			1.05	0.61***
Log likelihood			(0.03)	(0.07)
Prob > *χ*^2^	0.000	0.000	0.000	0.000

*p < 0.1; **p < 0.05; ***p < 0.01

^a^Centered in models with inteaction term. Clustered robust standard errors in parenthesis. No collinearity according to Variance Inflation Factors. Weight: share of number of applicant countries

Actually, the main suspects of change after this modification do not alter their sign or significance: the odds ratio of filing year is still smaller than one and significant both for Euro-PCT and for EPOrg applications, and following a direct EPO procedure is significant for non-EPOrg members.

Results about the rest of variables also lead to similar findings as before. In particular, the odds ratios of grants and per capita GDP are always larger than one and significant, as is GERD intensity for direct EPO applications and EPOrg member states. Being an EPOrg member and, within EPOrg, coincidence between examiners’ and applicants’ nationality also present larger than one odds ratios and significant signs.^10^^,^^11^

Large sample sizes have many advantages, like a precise estimation of odds ratios, the possibility of including many control variables and interactions, and performing split sample analysis without losing precision. We have used these advantages so far. However, large sample sizes present the potential problem of deflating standard errors, so that significance levels may artificially increase. Notice that some odds ratios in the previous tables are not significant, so inflation is not systematically present in the data. Moreover, our theory backs up our findings, together with the previous robustness checks and the counterbalancing inflation of standard errors caused by country clustering. We have also presented odds ratios to discard statistical without practical significance (across estimations, one can see that effect sizes are considerable). Nevertheless, in order to further take into account the potential bias of using a large sample of several million observations, we have created a set of random subsamples of 10%, and 1% of the total sample and replicated our previous analyses. The results in Table 9 show that all of our hypotheses hold, regardless of the number of observations. Apart from the result shown, we have performed the same exercise with two other sets of random subsamples, and the findings are similar.^12^

**Table 9:** Logistic regression of the probability of examiner trust (an applicant originating a citation rather than the examiner) at the EPO: randomly reduced samples (odds ratios)

	1 10% sample	2 EPOrg 10% sample	3 1% sample	4 EPOrg 1% sample
Direct EPO^a^	1.17	0.94*	1.16**	0.98
	(0.16)	(0.03)	(0.10)	(0.05)
Grant	1.36***	1.35***	1.33***	1.32***
	(0.02)	(0.02)	(0.05)	(0.05)
Non-patent literature	0.96**	0.97***	0.96*	0.96***
	(0.02)	(0.01)	(0.02)	(0.01)
Filing year	0.93*	0.89**	1.02	0.89**
	(0.04)	(0.05)	(0.09)	(0.05)
B Perf. Op.; Transp.	1.80***	1.84***	1.75***	1.82***
	(0.10)	(0.11)	(0.13)	(0.09)
C Chemistry; Metallurgy	1.49***	1.44***	1.48**	1.33**
	(0.07)	(0.05)	(0.26)	(0.18)
D Textiles; Paper	0.86**	0.85***	0.89	0.86
	(0.06)	(0.04)	(0.11)	(0.10)
E Fixed Constructions	0.80***	0.73***	0.84	0.73***
	(0.06)	(0.05)	(0.09)	(0.05)
F Mechanical Eng.; etc.	0.73***	0.77***	0.71***	0.69***
	(0.04)	(0.05)	(0.04)	(0.07)
G Physics	0.61***	0.65***	0.64***	0.59***
	(0.06)	(0.06)	(0.06)	(0.07)
H Electricity	1.07	0.69***	1.13	0.62***
	(0.34)	(0.05)	(0.42)	(0.09)
University/Government	0.99	1.13	1.03	1.14
	(0.09)	(0.10)	(0.12)	(0.15)
Nr applications	0.00**	0.00***	0.00***	0.00***
	(0.00)	(0.00)	(0.00)	(0.00)
Per capita GDP	1.02***	1.02*	1.02***	1.02*
	(0.01)	(0.01)	(0.00)	(0.01)
GERD intensity^a^	1.15	1.02	1.15	0.92
	(0.11)	(0.10)	(0.10)	(0.09)
GERD intensity × Direct EPO	0.62***	0.80**	0.69***	0.72**
	(0.09)	(0.07)	(0.10)	(0.11)
EPOrg member	2.40***		2.23***	
	(0.20)		(0.18)	
EPO exam same country		2.06**		2.13*
		(0.74)		(0.95)
Observations	366,328	167,211	36,633	16,647
Log likelihood	− 83,640	− 47,869	− 8,499	− 4,752
Prob > *χ*^2^	0.000	0.000	0.000	0.000

*p < 0.1; **p < 0.05; ***p < 0.01

^a^Centered in models with inteaction term. Clustered robust standard errors in parenthesis. Weight: share of number of applicant countries

## Conclusions

In this paper, we endorse the idea that the analysis of examiner versus applicant citations still opens avenues to increase understanding about the complexity of the patent citation process (Jaffe and de Rassenfosse 2017). Previous work on citation probability of an applicant rather than an examiner citation in a patent provides little information on what this might mean. Alcácer et al. (2009) interpret it as indicating applicant search effort in the case of USPTO patents. In the EPO, at the citation level, we interpret it as indicating examiner trust in applicants. This takes account of the mediating role of examiners in the process of deciding which applicant references are relevant. Both qualitative evidence from interviews with agents in the EPO patent system and the positive association between probability of applicant citations and a patent being granted reinforce this interpretation. Our results suggest that large applicant citation probability is more clearly associated with being awarded a patent at the EPO than at the USPTO (Lemley and Sampat 2012; Cotropia et al. 2013).

Prior analyses of the characteristics of applicant citation probability found differences across application procedures, patent and applicant characteristics (Alcácer et al. 2009; Azagra-Caro et al. 2011; Park et al. 2017). We highlight the importance of other sources of variation: national characteristics of the applicant, specially in the light of interpreting applicant citations in EPO examiner reports as indicators of examiner trust. Trust is partly a social construct, shaped by national economic and scientific strengths, belonging to the same country bloc, and national matching between applicants and examiners.

Empirically, the use of a sample based on EPO applications allowed comparison with earlier work exploiting USPTO evidence. This shows that there are similar country bloc effects which favor US residents in the case of the USPTO (Alcácer el al. 2009), and EPOrg members in the case of the EPO. Since the methods used by Alcácer et al. (2009) and those applied in this study differ, interpretation of this comparison should be cautious. A possible avenue for further inquiry might be to design an experiment to enable a direct comparison between both data sources.

Another line of investigation exploits the distinction between applicant and examiner citations to search for potential weaknesses in examiner practices (Collins and Wyatt 1998; Meyer 2000; Lemley and Sampat 2012; Cotropia et al. 2013). These studies are mostly at the individual level, and do not allow straightforward comparison with our results. However, at the citation level, the present study offers large-scale quantitative evidence of national effects at the EPO. After controlling for differences in procedural aspects, technology fields, applicant characteristics, science relatedness, national economic strengths, etc., we find that the nationality of the applicant conditions examiner trust to EPOrg member countries. This suggests the presence of a systematic bias which is not attributable to the other factors we control for. This bias may exist despite good organizational practices at the EPO, and may be due to limitations in the cultural knowledge bases of EPO examiners. However, our analysis is limited by the use of a proxy and lack of data on the examiners of individual patent applications; we rely on national aggregates and indicators of the probabilities of finding an examiner from the same country. Qualitative research could provide additional support for this finding.

Finally, both in scientometrics and patentometrics, frequency of forward citations is a widespread indicator of value or technological impact. A systematic bias such as the one we identify would lead to noisy, unreliable indicators (Roach and Cohen 2013; Corsino et al. 2017). Several studies dealing with citations between patents already distinguish between applicant and examiner citations in order to address some of these biases. A further step could be done to reduce the noise of the citation analysis by differentiating examiner citations depending on the country of the citing patent.

## Footnotes

1 These studies tend to use the term ‘inventor citations’. We prefer ‘applicant citations’ because citations can originate in the inventor, or the applicant, or the person responsible for filing the patent application, e.g. the firm’s intellectual property rights manager, or an external patent attorney, but ultimate responsibility for the inclusion of citations is the applicants.

2 Concretely, Alcácer et al.’s (2009) study of the USPTO proposed applicant citation shares in patents indicate ‘applicant search effort’ rather than ‘applicant search capabilities’, based on the positive correlation between applicant self-citations and other citations.

3 We collected evidence from 15 people: 7 EPO examiners, 1 non-EPO European examiner, 4 European inventors and 2 agents of EPO applications, and 1 US inventor of an EPO application. They provided 10 oral interviews, 3 e-mail interviews, and 7 questionnaires. We contacted official sources at the EPO to gather the testimonies from its examiners, but did not get any reply (others have faced similar problems: see Callaert et al. 2010). EPO examiners’ views here are therefore personal and do not necessarily represent the official position of the organization.

4 Some studies define these categories in more detail (e.g. Meyer 2000).

5 We excluded patents without citations to prior documents. Some PCT patents have comments in Patstat such as ‘See references of WO 0046271A1’. The data for those references are accessible through the international application identifier field, and citations in that format were included in the study through the use of an algorithm of our own creation. However, due to an inconsistency in the Patstat database, some PCT patents do not have international identification numbers which meant we were unable to cross reference their data with their cited documents.

6 The starting year is 1997 because we lagged national economic and scientific variables 2 years and 1995 was the first year with available data on those variables. The ending year is 2007 for two reasons. First, our version of Patstat does not include citation categories after 2008. Second, we also excluded 2008 given the decrease in the number of applications, which is probably due to statistical, artificial reasons (delay induced by the grant process, delay in the update of the database, etc.).

7 A comparison between the sample and the population revealed that the huge drop was due to Euro-PCT and more recent applications, probably because of delays in the information update. We account for this by creating a stratified sample with the properties of the population at the robustness check sub-section of the results.

8 See full list of member states: http://www.epo.org/about-us/foundation/member-states.html [3/5/2018].

9 Table 6 shows some differences between the three largest patenting countries and other countries. The decrease in examiner trust is attributable only to the other countries. Technological field affects examiner trust to a lesser extent for the top three patenting countries. Scientific knowledge base and organizational mission have significant (and opposite) effects for other countries than the top three. The negative effect on examiner trust of companies that are frequent patent applicants applies to the top three patenting countries only.

10 Because this variable is a proxy, as a double check, we tested whether clustering is necessary. The result indicated no need for clustering. Specifically, for the probability of a match between applicant and examiner, the test rejects the null hypotheses of equality between the mean with country clustering (by groups of the variable *appcit*), and the combined mean without clustering (applying the facility developed by Herrin 2012). Estimations with robust, non-clustered standard errors report that the coefficient of the probability of an applicant finding an examiner from the same country has strong significance.

11 The results are also robust to the inclusion of national strength of patent protection, measured through data provided by Park (2008) and extrapolated to our panel in a fashion similar to Picci (2010).

12 We have not included here all replications for the sake of space. The results are available under request.
